# Disparities in menstrual hygiene management between urban and rural schoolgirls in Northeast, Ethiopia

**DOI:** 10.1371/journal.pone.0257853

**Published:** 2021-09-30

**Authors:** Bikis Yaynie Shibeshi, Amanu Aragaw Emiru, Melash Belacehew Asresie

**Affiliations:** 1 Department of Obstetrics and Gynecology Nefasmewucha Hospital, Amhara Regional State, Bahir Dar, Ethiopia; 2 Department of Reproductive Health and Population Studies, School of Public Health, College of Medicine and Health Sciences, Bahir Dar University, Bahir Dar, Ethiopia; University Medical Center Utrecht, NETHERLANDS

## Abstract

**Introduction:**

Even though menstruation is a normal biological process, adolescents are facing managing their menstruation when they are at school. It causes girls to miss their class on average three days every month. Studies in some countries showed that the magnitude of menstrual hygiene management problems is higher in rural adolescent girls, but little is known in the Ethiopia context. The objective of this study was to assess and compare menstrual hygiene management practices among rural and urban schoolgirls, Northeast, Ethiopia.

**Methods:**

An institution-based comparative cross-sectional study was employed among 1078 schoolgirls (539 urban and 539 rural) from February to March 2020. The participants were selected using a multi-stage sampling technique. A structured self-administrative questionnaire and observational checklist were used for data collection. Bivariate and multivariable logistic regression analysis with a 95% confidence interval was employed. A P- value less than 0.05 was used to declare statistical significance.

**Results:**

Overall, the magnitude of good menstrual hygiene practice was 52.9% (95%CI: 50.3%-56.5%), which was 65.9% (95% CI: 62.8%-70.7%) among urban and 39.9% (95% CI: 36.2%-44.6%) among rural schoolgirls. Among urban schoolgirls, the odds of good menstrual hygiene management practice was higher for girls aged below 18 years (AOR = 1.58, 95%CI: 1.05–2.39), learned about menstrual hygiene at school (AOR = 1.89, 95%CI: 1.21–2.97), heard about menstrual hygiene before menarche (AOR = 4.98, 95%CI: 2.71–9.13), and discussed menstrual hygiene with parents (AOR = 2.56, 95%CI: 1.25–5.27). Whereas, the odds of good menstrual hygiene management practice was higher among those who were knowledgeable on menstrual hygiene (AOR = 5.47, 95%CI: 3.68–8.12), those who learned about menstrual hygiene at school (AOR = 1.75, 95%CI: 1.13–2.70), and girls who heard about menstrual hygiene before menarche (AOR = 3.34, 95%CI: 1.44–7.76) in rural schoolgirls.

**Conclusions:**

Though the overall menstrual hygiene practice was low, it was relatively better among urban schoolgirls. This calls for more effort to solve these problems and achieve sustainable development goals. Therefore, education and awareness creation on menstrual hygiene for schoolgirls, even before menarche at both settings should be strengthened. Encouraging parent-adolescent discussion on menstrual hygiene would have paramount importance, particularly to urban schoolgirls.

## Introduction

Adolescents (10–19 years) make up 16% of the world population and 23% of the Sub-Saharan Africa population. An adolescent is a critical time of rapid physical, psychological, and cognitive changes that affect how they feel, think, make the decision, and interact with the world around [1–4].

Menarche is a developmental milestone and is a transition period of girls from childhood to womanhood that happens during the adolescent period [5]. It is the foundation of menstruation, which is part of the female reproductive cycle that starts when girls become sexually mature during puberty [6].

Menstrual hygiene management (MHM) refers to personal hygiene during menstruation, which includes bathing, using sanitary pads and change pads regularly, and proper disposal of used materials [7]. A safe and effective menstrual hygiene management is a trigger for better development for adolescent girls, equipping them with knowledge and skills on MHM, which enhance their self-esteem, and positively impact their academic performance [8]. In this regard, safe MHM will play a role in the achievement of the sustainable development goals (SDG), including; good health and well-being (SDG 3), inclusive and impartial quality education (SDG 4), gender equality, and women’s empowerment (SDG 5), clean water and sanitation (SDG6) [9,10]. Yet, many adolescents are facing challenges in managing their menstruation at school especially in low and middle-income countries [9].

Poor MHM negatively influences the health, dignity, and privacy of millions of girls [11,12]. Menstrual hygiene-related problems have negative impacts on girl’s lifestyle, health, and developmental opportunities including absence from their school. Girls may also become worried due to consequences of poor MHM including, offensive smell, symptoms of reproductive tract infection such as itching of the vulva, pain during urination, and vaginal discharge [13–15].

In low and middle-income countries, MHM is a challenge for many girls mainly when attending school [6]. Girl’s education is a foundation of development and an entry point to many activities including politically, economically, socially participation. However, globally schoolgirls miss their classes during menstruation. In Bangladeshi, for example, about 38% of the urban schoolgirls and 42% of the rural schoolgirls reported missing classes during menstruation [12]. In India, one out of four girls is absent to attend school during the menstrual period [16]. Other studies conducted in India showed the proportion of good MHM practice among urban adolescents was almost two times compared to rural girls [17,18].

In Africa, studies revealed that 50% up to 70% of girls missed on average 1.6–2.1 days from school every month due to menstrual-related issues such as shame, pain, and uncomfortable among others [19,20]. In Ethiopia, about 43% up to 51% of students missed their class during the menstruation period [21,22]. Despite this huge problem, MHM did not receive the attention of educational policies and countries’ school curriculums in low and middle-income countries [23].

Studies in other countries have depicted that the magnitude of menstrual hygiene problems was higher in rural adolescent girls [17,18,24]. Whereas awareness of menstrual hygiene management among rural adolescents was low[18]. This indicates that urban-rural and area-based studies are important to understand the level of problems and develop evidence-based strategies to tackle the MHM-related problems. There are some studies conducted in Ethiopia among school adolescent girls [25–27], but didn’t analysis and interpret by urban and rural settings. In Ethiopia, there is cross-sociocultural diversity among the urban and rural community which affect mensural hygiene management practice. As the authors are aware there is one comparative study conducted in Ethiopia among urban and rural girls [28], but it was a community-based study that didn’t show the problem of MHM practice at school. Because of this, we believe that there is limited information about the proportion of good MHM practice and determinants for variation among adolescent girls by residences in Ethiopia.

Therefore, this study aimed to assess menstrual hygiene management practice and its associated factors among urban and rural schoolgirls in Northeast Ethiopia.

## Materials and methods

### Study area and period

This study was carried out in North Wollo Zone from February to March 2020. North Wollo is located in Amhara National Regional State, Northeast Ethiopia. Woldia is the capital city of North Wollo Zone, far about 521km from Addis Ababa, the capital city of Ethiopia. The current estimated population of the Zone is 1,824,361 of these 910,789 are females. In the Zone, there are 53 (15 urban and 38 rural) secondary schools. The total grade nine and grade ten high schoolgirls registered in the 2019/2020 academic year were 40,528. Of these, 14,564 students were females. Of female students, 7,573(52%) were enrolled to urban schools [29].

### Study design and study population

An institution-based comparative cross-sectional study was conducted among urban and rural high school female students (grade 9 and grade 10).

### Sample size determination

The minimum sample size for each group (rural and urban) was calculated using the formula for the two-sample comparison of proportion by considering the following assumptions: 95% confidence interval, 80% power, and prevalence of good MHM among urban (94%) and rural (86.5%) schoolgirls from a previous study [30]. Adding a 10% non-response rate and considering the designing effect of 2, the calculated final sample size became 1078 (539 urban; 539 rural).

### Sampling procedure and technique

A multi-stage sampling technique was used to select the study participants. First, 3 out of 15 urban schools and 8 out of 38 rural high schools were selected using a lottery method simple random sampling technique. Second, after proportional allocation had been done to the selected schools at each setting, thirty percent of grade nine and grade ten sections were selected using a simple random sampling technique. Finally, study participants were selected by a simple random sampling technique again.

### Definitions and measurements

#### Knowledge of menstrual hygiene

A girl was considered as knowledgeable on MHM, if she scored mean or above the mean from knowledge-related questions. Each girl was asked 9 knowledge-related questions concerning MHM and each correct answer was given a value of “1” if she answered correctly and a value of “0” otherwise [31].

#### Good menstrual hygiene management practice

A girl was considered as having good menstrual hygiene management practice, if she scored mean or above the mean from practice-related questions, otherwise considered as having poor menstrual hygiene management practice. Each girl was asked nine practice-related questions concerning MHM and each correct answer was given a value of “1” if she answered correctly and a value of “0” otherwise [31].

#### Functional toilet compartment

Toilets were considered functional when they are not physically broken and were actively used at the time of the survey.

#### Partially functional toilet compartment

Toilets were considered partially functional, if the toilets were still used despite there were at least some problems with the physical infrastructure including deterioration in concrete, loose doors, and deteriorating roof among others [32].

### Data collection tools and procedures

A pretested self-administered questionnaire was adapted from literature [30,31,33,34]. An observational checklist was also used to assess the presence of separate toilets for females versus males, the presence of water around the toilet, cleanness of the toilet, and other related issues. The questionnaire was prepared in English then translated into the local language (Amharic) and re-translated back to English to check its consistency. After receiving a two-day intensive training, eight female diploma midwives and four bachelor holder midwives were deployed as data collectors and supervisors, respectively. Close supervision was made on daily basis.

### Data processing and analysis

The collected data were entered using epi-info version 7 and transported to SPSS version 23 for analysis. Both descriptive and analytical statistics were computed. Both bivariate and multivariable logistic regression analyses were done to identify factors associated with menstrual hygiene management practice for each group. The explanatory variables with p<0.25 in the bivariate analysis were entered into multivariable analysis to control the effect of confounders. Before conducting independent logistic regression analysis for urban and rural school girls, chi-square testing was done to see if there was any significant difference in the prevalence of good MHM practice among the two groups [35]. Adjusted odds ratios (AOR) with 95% CI was calculated to measure the strength of association, and a p-value < 0.05 was considered statically significant. Model fitness was tested by using the Hosmer-Lemeshow Goodness of fit test. It was well fitted (p = 0.89 and 0.64 among urban and rural girls, respectively).

### Ethical approval and consent to participate

Ethical clearance was obtained from the ethical review board of Bahir Dar University. Permission letter was also secured from the North Wollo Zonal education bureau, Woreda education offices, and schools. The aim of the study was informed for each study participant and the study participants were informed about their right to refuse or discontinue participating in the research without any restriction. After explained the aim and purpose of the study, informed consent was obtained from all subjects or, if subjects are under 18 years, from a parent and/or legal guardian. Confidentiality and privacy were assured by excluding personal identifiers. All methods were carried out in accordance with relevant guidelines and regulations.

## Results

### Socio-demographic characteristics

Five hundred and thirty-nine girls from each group (urban and rural) participated in this study. The mean (±SD) age of urban and rural schoolgirls was 17.2 (±1.3) and 17.5 (±1.6) years, respectively. The highest proportion, 395(73.3%) of urban and 309(57.3%) of rural schoolgirls were below 18 years. The mean(±SD) age of menarche was 13.3 (±1.10) and 13.89 (±1.03) years in urban and rural schoolgirls, respectively. Four hundred and sixty-three (85.9%) of the urban and 514(95.4%) of rural schoolgirls were Orthodox Christian followers (Table 1).

**Table 1:** Socio-demographic characteristics of urban and rural schoolgirls in Northeast, Ethiopia, 2020 (N = 1078: 539 urban and 539 rural).

Variables	Category	Urban No (%)	Rural No (%)	Total No (%)
Age in year				
	<18	395 (73.3)	309 (57.3)	704 (65.3)
	≥18	144 (26.7)	230 (42.7)	374 (34.7)
Grade				
	9	395 (73.3)	340 (63.1)	735 (68.2)
	10	144 (26.7)	199 (36.9)	343 (31.8)
Religion				
	Orthodox	463 (85.9)	514 (95.4)	977 (90.6)
	Muslim	36 (6.7)	15 (2.8)	51 (4.7)
	Protestant	40 (7.4)	10 (1.9)	50 (4.7)
Marital status				
	Single	504 (93.5)	495 (91.8)	999 (92.7)
	Married	22 (4.1)	28 (5.2)	50 (4.6)
	Divorced	13 (2.4)	16 (3.0)	29 (2.7)
Ethnicity				
	Amhara	519 (96.3)	511 (94.8)	1030 (95.5)
	Others*	20 (3.7)	28 (5.2%)	48 (4.5)
Educational status of mothers^,^				
	Not attending formal education	44 (8.2)	390 (72.4)	434 (40.3)
	Primary school	67 (12.4)	72 (13.3)	139 (12.9)
	Secondary school or above	428 (79.4)	77 (14.3)	505 (46.8)
Educational status of fathers’(n = 1057)				
	Not attending formal education	88 (16.5)	433 (82.8)	521 (49.6)
	Primary school	34 (6.4)	66 (12.6)	100 (9.5)
	Secondary school or above	412 (77.1)	24 (4.6)	436 (40.9)
Occupational status of mothers^’^				
	Housewife	94 (17.4)	346 (64.2)	440 (40.8)
	Daily laborer	17 (3.2)	89 (16.5)	106 (9.8)
	Merchant	149 (27.6)	71 (13.2)	220 (20.4)
	Government employed	214 (39.7)	18 (3.3)	232 (21.6)
	Private organization	65 (12.1)	15 (2.8)	80 (7.4)
Occupational status of Fathers’(n = 1057)				
	Farmer	22 (4.1)	294 (56.2)	316 (29.9)
	Daily laborer	113 (21.2)	164 (31.4)	277 (26.2)
	Merchant	210 (39.3)	41 (7.8)	251 (23.7)
	Government Employed	161 (30.1)	12 (2.3)	173 (16.4)
	Private organization	28 (5.2)	12 (2.3)	40 (3.8)

**Key**: Other* Tigre, Oromo.

### Information related to menstrual hygiene

The majority of urban 494 (91.7%) and rural 483(89.6%) schoolgirls heard about menstrual hygiene before the onset of menarche. Mothers were the main source of information for 359 (66.6%) urban and 104 (19.3%) rural girls. The findings indicated that eight in ten (80.0%) of the urban and nearly seven for every ten (69.0%) of the rural schoolgirls learned MHM issues at their schools. Almost, similar proportion, which was of the urban 504 (93.5%) and rural 502(93.1%) schoolgirls reported that they were openly discussing menstrual hygiene with their parents.

### School environment-related issues

Though all schools in the urban and rural setups had separate latrines for girls and boys, they were lacking handwashing facilities inside the latrine and were not clean enough to change menstrual materials; there were some smells and/or some signs of fecal matter/urine and/or some flies) around the seats. In relation to this, only 147 (27.3%) of urban and 98(18.2%) of rural girls responded that school latrine was comfortable to change menstrual materials. Regrettably, only 67(12.4%) of the urban and 36(6.7%) of the rural schoolgirls were accessing water in the school compounds to keep personal hygiene.

### Knowledge about menstrual hygiene

Overall, 670 (62.2%) of schoolgirls (74.4% among urban and 49.9% rural girls) were knowledgeable about menstrual hygiene. Four hundred twenty-nine (79.6%) of the urban and 158(29.3%) of the rural schoolgirls reported menstruation as a physiological process, while only 7(1.3%) of the urban and 15(2.8%) of the rural schoolgirls had no idea about the cause of it **(**Table 2).

**Table 2:** Knowledge about menstrual hygiene among urban and rural schoolgirls in North Wollo Zone, Northeast Ethiopia, 2020 (N = 1078; 539 urban and 539 rural).

Variables		Urban N (%)	Rural N (%)	Total N (%)
What is menstruation?				
	Physiological process*	429 (79.6)	394 (73.1)	823 (76.3)
	Curse from God	75 (13.9)	95 (17.7)	170 (15.8)
	Pathological process	28 (5.2)	35 (6.5)	63 (5.9)
	Don’t know	7 (1.3)	15 (2.8)	22 (2.0)
Source of menstrual blood				
	Vagina	78 (14.5)	108 (10.1)	186 (17.3)
	Urinary bladder	8 (1.5)	12 (2.2)	20 (1.9)
	Uterus*	432 (80.1)	372 (69.1)	804 (74.6)
	Don’t know	21 (3.9)	47 (8.7)	68 (6.3)
Normal menstrual bleeding duration				
	< 2 days	21 (3.9)	50 (9.3)	71 (6.6)
	2 to 7 days*	444 (82.4)	344 (63.8)	788 (73.1)
	> 7 days	65 (12.1)	112 (20.8)	177 (16.5)
	Don’t know	9 (1.7)	33 (6.1)	42 (3.9)
Normal menstrual cycle				
	< 21 days	42 (7.8)	50 (9.3)	92 (8.6)
	21 to 35 days*	400 (74.2)	344 (63.8)	744 (69.0)
	> 35 days	83 (15.4)	112 (20.8)	195 (18.1)
	Don’t know	14 (2.6)	33 (6.1)	47(4.4)
Taking more nutrition during menses				
	Yes*	420 (77.9)	345 (64.0)	765 (71.0)
	No	119 (22.1)	194 (35.9)	313 (29.0)
Washing perineum during menses				
	Yes*	442 (82.0)	407 (75.5)	849 (78.8)
	No	97 (18.0)	132 (24.5)	229 (21.3)
Bathing doesn’t cause infertility				
	Yes*	448 (83.1)	424 (78.7)	872 (80.9)
	No	91 (16.9)	115 (21.3)	206 (19.1)
Painkillers take during painful menses				
	Yes*	297 (55.1)	151 (28.0)	448 (41.6)
	No	242 (44.9)	388 (72.0)	630 (58.5)
Can poor hygiene predispose to infection				
	Yes*	346 (64.2)	287 (53.2)	633 (58.7)
	No	193 (35.8)	252 (46.8)	445 (41.3)
overall knowledge				
	Good	401 (74.4)	269 (49.9)	670 (62.2)
	Poor	138 (25.6)	270 (50.1)	408 (37.8)

Key

* was code as “1” and the rest were coded as “0”.

### Menstrual hygiene management practice

The overall prevalence of good menstrual hygiene management practice was 52.9% (95%CI: 50.3%-56.5%), which was 65.9% (95% CI: 62.8%-70.7%) among urban and 39.9% (95% CI: 36.2%- 44.6%) among rural schoolgirls (p<0.001). Four hundred and nine (88.9%) of the urban and 285(54.7%) of the rural girls were used commercial sanitary pads. The majority, 475 (88.1%) of urban and 455(84.4%) of rural school girls clean their external genitalia during menstruation. Almost a similar proportion, which was of the urban 448(83.1%) and the rural 439(81.4%) school girls had a habit of changing sanitary material during menstruation (Table 3).

**Table 3:** Menstrual hygiene practice among urban and rural schoolgirls in Northeast, Ethiopia, 2020 (N = 1078; 539 urban and 539 rural).

Variables		Urban n (%)	Rural n (%)	Total n (%)
Use any sanitary materials/pads				
	Yes	539 (100.0)	521 (96.7)	1060 (98.3)
	No	0 (0.0)	18 (3.3)	18 (1.7)
Use commercial pads (n = 1060)				
	Yes	479 (88.9)	285 (54.7)	764 (72.1)
	No	60 (11.1)	236 (45.3)	296 (27.9)
Clean external genitalia during menstruation				
	Yes	475 (88.1)	455 (84.4)	930 (86.3)
	No	64 (11.9)	84 (15.6)	148 (13.7)
Cleans external genitalia with water and soap during menstruation (n = 930)					
	Yes	335 (70.5)	142 (31.2)	477 (51.3)
	No	140 (29.5)	313 (68.8)	453 (48.7)
Clean external genitalia at least three times per day (n = 930)*				
	Yes	342 (72.0)	201 (44.2)	543 (58.4)
	No	133(28.0)	254(55.8)	387(4.6)
Taking bath with soap at least once per day			
	Yes	449 (83.3)	224 (41.6)	673 (62.4)
	No	90 (16.7)	315 (58.4)	405 (37.6)
Changing sanitary material during menstruation				
	Yes	448 (83.1)	439 (81.4)	887 (82.3)
	No	91 (16.9)	100 (18.6)	191 (17.7)
Changing sanitary pads or cloths at least three times per day during menstruation				
	Yes	326 (72.8)	15 (3.4)	341 (38.4)
	No	122(27.2)	424 (96.6)	546 (61.6)
Dispose of used sanitary pads in the dustbin				
	Yes	98 (21.9)	39 (8.9)	137 (15.4)
	No	350 (78.1)	400 (91.1)	750 (84.6)
MHM Practice				
	Good	355 (65.9)	215 (39.9)	570 (52.9)
	Poor	184 (34.1)	324 (60.1)	508 (47.1)

### Factors associated with MHM practice among school girls

As stated in the methods section, chi-square testing was done to see if there was any significant difference in the prevalence of good MHM practice between urban and rural school girls. Accordingly, a statistically significant difference was observed between the two groups (x^2^ = 72.97, df = 3, p<0.001. Moreover, in the bivariate (COR = 2.91(2.27, 3.73) and multivariable (AOR = 1.77(1.18, 2.65) analyses, the type of school was significantly associated with MHM practice suggesting differences in MHM practice between urban and rural school girls. Therefore, the analysis was conducted separately for urban and rural school girls.

### Factors associated with MHM practice among urban schoolgirls

Age of girls, discussions with parents about MHM, awareness on MHM before the onset of menarche, and learned about menstrual hygiene at school were significantly associated with MHM practice in the multivariable analysis. Schoolgirls below 18 years of and those who learned about menstrual hygiene at the school level were almost twice more likely to have good MHM practice as compared to their counterparts (AOR = 1.58, 95%CI = 1.05, 2.39) and (AOR = 1.89, 95%CI = 1.21, 2.97), respectively. The odds of good MHM practice among schoolgirls who heard about menstrual hygiene before the onset of menarche was 4.98 higher compared to their counterparts (AOR = 4.98, 95%CI = 2.71, 9.13). Those girls who did have an open discussion with parents on issues related to MHM were 2.56 more likely as compared to schoolgirls who did not have an open discussion about menstrual hygiene with their parents (AOR = 2.56, 95% CI = 1.25, 5.27) (Table 4).

**Table 4:** Factors associated with menstrual hygiene management practice among urban schoolgirls in Northeast, Ethiopia, 2020 (n = 539).

Variable	Menstrual hygiene practice		COR, 95%CI	AOR, 95%CI
	Good n (%)	Poor n (%)		
Age				
<18	272 (50.5)	123 (22.8)	1.63 (1.09, 2.41)	1.58 (1.05, 2.39) *
≥18	83 (15.4)	61 (11.3)	1	1
Learned about menstrual hygiene at school				
Yes	296 (54.9)	135(25.0)	1.82 (1.19, 2.79)	1.89 (1.21, 2.97) **
No	59 (10.9)	49(9.1)	1	1
School water comfortable to keep menstrual hygiene				
Yes	38 (7.1)	29 (5.4)	0.64 (0.38, 1.08)	0.66 (0.35, 1.24)
No	317(58.8)	155 (28.8)	1	1
Heard about menstrual hygiene before menarche				
Yes	337 (62.5)	146 (27.1)	4.87 (2.69, 8.82)	4.98 (2.71, 9.13) ***
No	18 (3.3)	38 (7.1)	1	1
School latrine comfortable to change sanitary materials				
Yes	90 (16.7)	57 (10.6)	0.76 (0.51, 1.12)	0.92 (0.57, 1.49)
No	265 (49.2)	127 (23.6)	1	1
Discussed menstrual hygiene with their parents				
Yes	339 (62.9)	165(30.6)	2.44 (1.22, 4.87)	2.56 (1.25, 5.27) *
No	16 (3.0)	19 (3.5)	1	1

**Key**

* P-value <0.05

** p-value <0.01

*** p-value <0. 001, COR = Crude Odds Ration, AOR = Adjusted Odds Ration, CI = Confidence interval, n = frequency.

### Factors associated with MHM practice among rural schoolgirls

Knowledge of menstrual hygiene, awareness of MHM before the onset of menarche, and learned about menstrual hygiene at school were significantly associated with good practice of MHM in the multivariate analysis. Schoolgirls who were knowledgeable about MHM were 5.47 more likely to have good MHM practice as compared to their counterparts (AOR = 5.47, 95%CI = 3.68, 8.12). The odds of good MHM practice among schoolgirls who learned about menstrual hygiene at school was 1.75 higher compared to schoolgirls who didn’t learn about menstrual hygiene (AOR = 1.75, 95%CI = 1.13, 2.70). Schoolgirls who awarded/heard about MHM before attaining their menarche were more likely to have good MHM practice as compared to their counterparts (AOR = 3.34, 95%CI = 1. 44, 7.76) (Table 5).

**Table 5:** Factors associated with menstrual hygiene management practice among rural schoolgirls in Northeast, Ethiopia, 2020 (n = 539).

Variables	Menstrual hygiene practice		COR, 95%CI	AOR, 95%CI
	Good n (%)	Poor n (%)		
Age				
<18	132 (24.5)	177 (32.8)	1.32 (0.93, 1.88)	1.03 (0.69, 1.54)
≥18	83 (15.4)	147 (27.3)	1	1
Educational status of mothers^,^				
Secondary or above	39 (7.2)	38 (7.1)	1.75 (1.07, 2.87)	1.43 (0.82, 2.48)
Primary	32 (5.9)	40 (7.4)	1.37 (0.82, 2.27)	1.71 (0.96, 3.05)
No formal education	144 (26.7)	246 (45.6)	1	1
Knowledge on MHM				
Good	158 (29.3)	111 (20.6)	5.32 (3.64, 7.78)	5.47 (3.68, 8.12) **
Poor	57 (10.6)	213 (39.5)	1	1
School water-access comfortable to keep menstrual hygiene				
Yes	11 (2.0)	25 (4.6)	0.65 (0.31, 1.34)	1.02 (0.43, 2.44)
No	204 (37.8)	299 (55.5)	1	1
Learned menstrual hygiene at school				
Yes	166 (30.8)	206 (38.2)	1.94 (1.31, 2.87)	1.75 (1.13, 2.70) *
No	49 (9.1)	118 (21.9)	1	1
School latrine comfortable to change sanitary materials				
Yes	32 (5.9)	66 (12.2)	0.68 (0.43, 1.09)	0.62 (0.36, 1.08)
No	183 (34.0)	258 (47.9)	1	1
Heard about menstrual hygiene before menarche				
Yes	207 (38.4)	287 (53.2)	3.34 (1.52, 7.31)	3.34 (1.44–7.76) **
No	8 (1.5)	37 (6.9)	1	1
Discussing menstrual hygiene with their parent				
Yes	207 (38.4)	295 (54.7)	2.54 (1.14, 5.68)	2.03 (0.84, 4.88)
No	8 (1.5)	29 (5.4)	1	1

**Key**

* P-value <0.05

** p-value <0.01

*** p-value <0. 001, COR = Crude Odds Ration, AOR = Adjusted Odds Ration, CI = Confidence interval, n = frequency.

## Discussion

The overall prevalence of good menstrual hygiene management practice among schoolgirls was 52.9% (95%CI: 50.3%-56.5%), higher compared with the studies done in Bahir Dar (24.5%) [28] and western Ethiopia (39.9%) [31]. The reason for variation could be explained as the sociodemographic difference of study participants, about 18% and 36.4% of participants in the study Bahir Dar and western Ethiopia were didn’t attend formal education, and 67% of participants in the study Bahir Dar were rural residents, negatively affect good MHM practice [36]. However, it was lower compared to studies done in Bahir Dar (84.3%) [37], Mehalmeda (90.9%) [30], and Batu (66.8%) [38]. It could be explained that most of the participants in the above studies were urban residents, which enable them to access sanitary materials. Besides Bahir Dar and Mehalmeda’s studies had included grade 11 and 12 schoolgirls, which may positively affect practicing of good MHM.

The prevalence of good menstrual hygiene management practice among urban schoolgirls was 65.9% (95% CI: 62.8%-70%.7) higher compared to 39.9% (95% CI: 36.2%- 44.6%) among rural schoolgirls, which was made statistically significant (p<0.001). The urban-rural difference in MHM practice was reported from the studies done in other countries [17,18,24,39]. The reason for variation in menstrual hygiene management between urban and rural in Ethiopia might be the accessibility of sanitary materials to keep menstrual hygiene, which is more accessible and available in an urban setting. The other reason might be a sexual and reproductive discussion with parents is high in urban settings, which may enable them to be knowledgeable and pre-informed on mensural hygiene. Urban girls have better exposure to media, increasing the chance to acquire health information and communication on menstrual hygiene management practices than rural girls. However, study done in Bahir didn’t show significant variation between urban and rural adolescents, it was 29.5% among urban schoolgirls and 21.9% among the rural schoolgirls. In addition, it was very low compared to the current study in both settings [28]. It could be explained that 18% of participants in the study Bahir Dar were not attended formal educations negatively affect MHM practice. The other might be due to enhancement in accessibility and availability of sanitary pads, improvement in the understanding of schoolgirls about menstrual hygiene management, enrichment in water, and sanitary facilities in the school environment.

On multivariable logistic regression analysis, heard about MHM before the onset of menarche and learned about MHM at school were associated with MHM practice at both settings. Besides, the age of girls and discussion on MHM in urban schoolgirls and Knowledge of MHM in rural schoolgirls were significantly associated with Good MHM practice.

Rural schoolgirls who were knowledgeable on menstrual hygiene were more likely to practice good menstrual hygiene as compared to their counterparts. This finding was supported by studies conducted in Wogera [33], Mehalmeda [30], Gedeo [40], and Ghana [41]. The reason for this might be the more they are aware of menstrual hygiene, the more likely they will develop confidence. However, it didn’t associate MHM practice among urban schoolgirls, might be the majority of urban schoolgirls are committed to it.

The current study also, reveals that urban schoolgirls aged below 18 years were more likely to practice good menstrual hygiene compared to schoolgirls aged 18 or above years. This finding was supported by studies conducted in Ambo [42] and Iran [43]. This could be explained that most of the time younger age girls are more likely to be supported by family, enable them to practice good menstrual hygiene [44], yet didn’t associate with rural schoolgirls. The reason might be most rural mothers are not using a sanitary pad, they may not encourage their daughters to practice good menstrual hygiene.

Urban schoolgirls who had an open discussion with parents about menstrual hygiene were more likely to practice good MHM practices than their counterparts. This result was supported by studies conducted in Addis Ababa [45] and Ambo [42]. The reason for this might be the more they discuss menstrual hygiene and other sexual and reproductive health issues, the more likely they will develop confidence and get support from their families, and also easy for schoolgirls to get hygiene-related materials without frustration.

Being learned about menstrual hygiene in the school, and hearing about menstrual hygiene before the onset of menarche were found to be associated with good MHM practice among both urban and rural schoolgirls, was agreed with other studies done in Addis Ababa [45], Ambo [42], and Mehalmeda [30]. The reason might be schoolgirls who are exposed to information regarding menstrual hygiene may increase their knowledge and confidence in menstrual hygiene management. The other reason might be sexual and reproductive education enables them to easily appreciate the potential threat and complication of the poor menstrual hygiene management.

The strength of this study was that, showed what looks menstrual hygiene management practice among urban and rural schoolgirls using separate sample sizes for each. The other strength was some observational information, not collected by other studies such as water and toilet availability and WASH facilities that could affect the menstrual hygiene management practice are reported. The limitation of this study was that the cross-sectional nature of this study may not show a causal-effect relationship between the outcome and explanatory variables.

## Conclusions

The overall good menstrual hygiene practice was low, it was relatively higher among urban schoolgirls than rural residents. There were disparities and similarities in factors of menstrual hygiene practice among the two settings. Learned about menstrual hygiene at school and being aware of the subject before the menarche were common factors both in urban and rural school girls. Among urban schoolgirls; age and discussed menstrual hygiene with their parents were also significantly associated with Good MHM. Knowledge on menstrual hygiene also associated with good MHM practice in rural schoolgirls.

This calls for more effort from the stakeholders to solve these problems and achieve sustainable development goals (goal 3, 4, 5, and 6). Therefore, strengthening adolescent girls’ education and awareness creation about menstrual hygiene at school even before menarche may improve good menstrual hygiene management practice among schoolgirls in both settings. In addition, encouraging open discussions with families on menstrual hygiene, particularly urban schoolgirls, may also improve menstrual hygiene management. At any effort, more emphasis should be given to rural school girls.

## Supporting information

S1 File (DOCX)
