# Supplementary material for: Disparities in menstrual hygiene management between urban and rural schoolgirls in Northeast, Ethiopia
Source: PLoS One. 2021 Sep 30;16(9):e0257853. doi: 10.1371/journal.pone.0257853 (PMC8483289; doi:10.1371/journal.pone.0257853)
Supplement: S1 File — (DOCX) [file pone.0257853.s001.docx]

**Measurements**

The mean (m) and standard deviation (SD) were calculated using the following formula: m=(∑x_i_)/n and SD=∑(x_i_-m)^2^/n where m=mean; x_i=_ the frequency of the i^th^; n=number of participants. The mean of age participants, age of menarche, knowledge of menstrual practice, and menstrual hygiene management practice was calculated using the above formula.

The age of the participant among urban schoolgirls ranged from 15-20 years, and among rural schoolgirls, it ranged from 15-23 years. Accordingly, the mean (+SD) age of urban and rural schoolgirls was 17.2 (±1.3) and 17.5 (±1.6) years, respectively.

The Knowledge of menstrual hygiene and menstrual hygiene management practice score was ranged from 0-9 for individuals. Every nine questions have two options; yes (1) and no (0). If they scored mean or above the mean from the knowledge of menstrual hygiene and menstrual hygiene management practice-related questions were grouped to knowledgeable and having good menstrual hygiene management practice, respectively. The overall mean of knowledge of menstrual hygiene was 6.2. Of urban and rural schoolgirls, 74.4% and 49.9% were scored the mean and above the mean, respectively. The overall mean of menstrual hygiene management practice was 5.4. Those who scored the mean and above the mean were 65.9% and 39.9% in the urban and rural schoolgirl participants, respectively.
